# Evaluation of U-Net models in automated cervical spine and cranial bone segmentation using X-ray images for traumatic atlanto-occipital dislocation diagnosis

**DOI:** 10.1038/s41598-022-23863-w

**Published:** 2022-12-12

**Authors:** Jae-Hyuk Shim, Woo Seok Kim, Kwang Gi Kim, Gi Taek Yee, Young Jae Kim, Tae Seok Jeong

**Affiliations:** 1 Department of Biomedical Engineering, Gil Medical Center, Gachon University College of Medicine, Incheon, Korea; 2 Department of Traumatology, Gil Medical Center, Gachon University College of Medicine, Incheon, Korea; 3 Department of Neurosurgery, Gil Medical Center, Gachon University College of Medicine, Incheon, Korea

**Keywords:** Trauma, Bone imaging

## Abstract

Segmentation of the cervical spine in tandem with three cranial bones, hard palate, basion, and opisthion using X-ray images is crucial for measuring metrics used to diagnose traumatic atlanto-occipital dislocation (TAOD). Previous studies utilizing automated segmentation methods have been limited to segmenting parts of the cervical spine (C3 ~ C7), due to difficulties in defining the boundaries of C1 and C2 bones. Additionally, there has yet to be a study that includes cranial bone segmentations necessary for determining TAOD diagnosing metrics, which are usually defined by measuring the distance between certain cervical (C1 ~ C7) and cranial (hard palate, basion, opisthion) bones. For this study, we trained a U-Net model on 513 sagittal X-ray images with segmentations of both cervical and cranial bones for an automated solution to segmenting important features for diagnosing TAOD. Additionally, we tested U-Net derivatives, recurrent residual U-Net, attention U-Net, and attention recurrent residual U-Net to observe any notable differences in segmentation behavior. The accuracy of U-Net models ranged from 99.07 to 99.12%, and dice coefficient values ranged from 88.55 to 89.41%. Results showed that all 4 tested U-Net models were capable of segmenting bones used in measuring TAOD metrics with high accuracy.

## Introduction

Traumatic atlanto-occipital dislocation (TAOD) is a traumatic injury stemming from damage to the spinal area resulting in dislocation of the upper cervical spine and the skull base^1,2^. Due to the high-energy nature of the trauma often associated with the injury such as motor vehicle accidents, most patients who experience atlanto-occipital dislocation have a high likelihood of mortality^3^. In cases of patients that survive the injury, it is vital that the dislocation is diagnosed and treated rapidly to improve the success rate of stabilization^4,5^. Patients who experience TAOD often present debilitating symptoms such as unconsciousness, respiratory arrest, and in severe cases, sensory, motor, and neurological deficits^2^. However, there lies some difficulty in consistently diagnosing TAOD in patients without neurological deficits due to the nature of certain symptoms, which can be as vague as severe neck pain, or injury to other areas of the body masking pain in the cervical area, to being completely asymptomatic^2^. To appropriately diagnose TAOD before complications from delayed treatment occur, radiological methods using MRI, CT, and X-rays are used to screen patients suspected of TAOD^1,6^.

**Figure 1:**
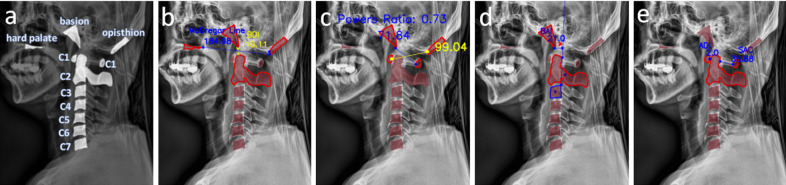
Example of metrics measured for TAOD diagnosis. (**a**) Bone structures of the cervical spine used for measuring TAOD diagnosing metrics. (**b**) McGregor line is a line that connects the posterior edge of the hard palate and the most caudal point of the opisthion. BDI is defined by the distance from the most inferior edge of the basion to the closest point of the superior edge of C2. (**c**) Power’s ratio, calculated by dividing the distance of the most inferior edge of the basion to the center edge of the right C1 by the distance of the leftmost point of the opisthion to the center point of the left C1. (**d**) BAI (line designated by the red arrow) is measured by the closest distance between the most inferior edge of the basion to the line that extends cranially along the posterior cortex of C2. (**e**) ADI refers to the closest distance between the center point of left C1 to the superior region of C2, while SAC refers to the closest distance between the center point of right C1 to the superior region of C2. All distances shown in this figure are in mm.

There are several guidelines used to diagnose TAOD through radiological methods^7,8^. Images of the cervical spine are taken either through MRI, CT, or X-rays, then studied for particular dislocations of specific cervical and cranial areas. One metric used to diagnose TAOD is the 10 mm increase in the basion-dens interval (BDI), which refers to the distance between the basion and the top of the C1 cervical bone by 10 mm^9^. Another metric is the Power’s ratio, which refers to the ratio of the distance between the opisthion to the anterior arch of C1, and the distance between the basion to the posterior arch of C1^10^. The basion-axial interval (BAI), which measures the distance between the basion and the C2 line is checked for a displacement of more than 12 mm or less than 4 mm for TAOD diagnosis^10^. The atlanto-dens interval (ADI), which refers to the distance between the anterior arch of the atlas and the dens of the axis, as well as space-available-cord (SAC), which refers to the distance between the posterior surface of the dens to the anterior surface of the posterior arch of the dens, are used to radiologically examine for TAOD^7^. McGregor line, which is a line that connects the hard palate to the most caudal point of the midline occipital curve, is also used to diagnose TAOD^11^. Examples of metrics measured for TAOD diagnosis are shown in Fig. 1. Depending on the injury sustained by the patient, each diagnostic method may show results with inconsistent conclusions, often requiring the clinician to use multiple techniques to assess for TAOD with sufficient confidence^6^.

With recent advances in CT and MRI imaging, high resolution, high contrast, 3D images of the cervical spine can be acquired with low scanning time. Compared to X-ray scans, CT and MRI scans can provide a much higher level of detail for accurately identifying key structures used to measure TAOD metrics such as BDI, BAI, and the power’s ratio^12^. However, there are certain constraints or situations when X-rays are recommended before CT or MRI scans are utilized. In the case of patients that report very mild symptoms of TAOD such as neck pain, it is likely that clinicians would recommend X-ray scans for cheap, fast, and accessible imaging of the cervical spine, especially if the clinic does not carry the necessary equipment for CT or MRI scans^12^. Additionally, X-rays may show sufficient resolution for identifying necessary metrics for TAOD diagnosis, making CT or MRI scans unnecessary^13^. As such, there is a benefit to developing methods for improving X-ray diagnosis of TAOD to ensure that the patients are rapidly diagnosed before complications of undiagnosed TAOD occur^6^.

Previous studies have explored methods to segment the cervical spine using X-rays. Early studies in segmenting the cervical spine drew references from spinal segmentation, which involved using techniques to obtain basic landmarks for a generalized location of the spine, then deforming a 2D contour model to match each individual vertebra based on boundary detection^14,15^. Various vertebrae corner and center detection algorithms were first used to establish the locations of each cervical vertebra^16,17^ then contours of each cervical vertebra were deformed to match the intensity of its reference using edge detection^18–20^. Machine learning approaches, such as Hough forest-based architectures and U-Net were adopted for a significantly stronger performing segmentation, being able to better discriminate between cervical vertebra and its surroundings^21–23^. However, previous studies on segmenting the cervical spine have been limited to bones up to C3, where outlines are clearly defined and unobstructed by the stacking effect of bones common in X-rays^22,23^. Certain aspects of the cervical spine make it difficult to delineate, some being the circular shapes of C1 and C2 bones causing stacking and the lack of clear boundaries for cranial bones like the opisthion or hard palate^22^.

For this study, we prepared a dataset of segmentations involving the cervical spine (C1 ~ C7) and three bones: opisthion, hard palate, and the basion. We then applied four different machine learning U-Net models: standard U-Net^24^, Attention U-Net (AU-Net)^25^, recurrent residual U-Net (R2U-Net)^26^, and Attention R2U-Net (AR2U-Net)^27^, then observed their performance metrics in the form of dice coefficient, sensitivity, specificity, and accuracy as well as model complexity through parameter count and floating-point operations per second (FLOPs). We quantitatively evaluated the segmentation metrics of each bone class between the 4 U-Net models in the form of dice coefficient, Hausdorff distance (HD) and mean square distance (MSD). Additionally, we qualitatively evaluated the common patterns of each U-Net model displayed in segmentations. Through our research, we aim to show that key bone structures involving in evaluating TAOD metrics can be automatically segmented using U-Net. Moreover, we aim to show unique patterns in segmentations that could arise utilizing each U-Net model based on the quantitative evaluation of segmentation metrics and qualitative evaluation of segmentation masks.

## Materials and methods

### Data acquisition

Sagittal X-rays of 707 subjects for delineation were collected at Gachon University Gil Medical Center. The average age of subjects was 53.9 (± 16.7), with 400 of them being male and 307 being female. Images had resolutions ranging from 0.139 mm per pixel to 0.194 and had an average of 1688 pixels as width and 2102 as height. All images were adjusted to face left, horizontally flipping X-rays of right-facing subjects. This study protocol was approved by the institutional review board at Gachon University Gil Medical Center (GDIRB2022-190). Methods used in this study were all in accordance with the relevant guidelines and regulations of the declaration of Helsinki, and informed consent was obtained from all participants involved in this study.

Contours of C1 ~ C7 bones along with the three bones, opisthion, hard palate, and basion, were delineated by two experts that received training from qualified medical doctors. An example of delineation is shown in Fig. 1a.

X-rays were resized to 512 × 512, and histogram equalized using contrast limited adaptive histogram equalization (CLAHE). 80% of the images (513 images) were randomly designated as the training and validation dataset. The remaining 20% (194 images) were used for testing the trained models.

### Network

The main framework for the three models used is U-Net, a commonly used convolutional neural network for the purpose of biomedical image semantic segmentation. U-Net consists of two pathways responsible for classifying and localizing each object in an image. The first pathway is referred to as the encoder, which involves multiple convolution, rectified linear unit (ReLU), and max-pooling layers to downsample data for feature extraction. The second pathway is referred to as the decoder, which involves up-sampling the extracted feature maps with convolution, concatenation, and ReLU layers for localization information. Through the encoder and decoder pathways, the U-Net architecture can efficiently extract both segmentation and location data, which enables low-cost training of highly accurate biomedical segmentation models.

**Figure 2:**
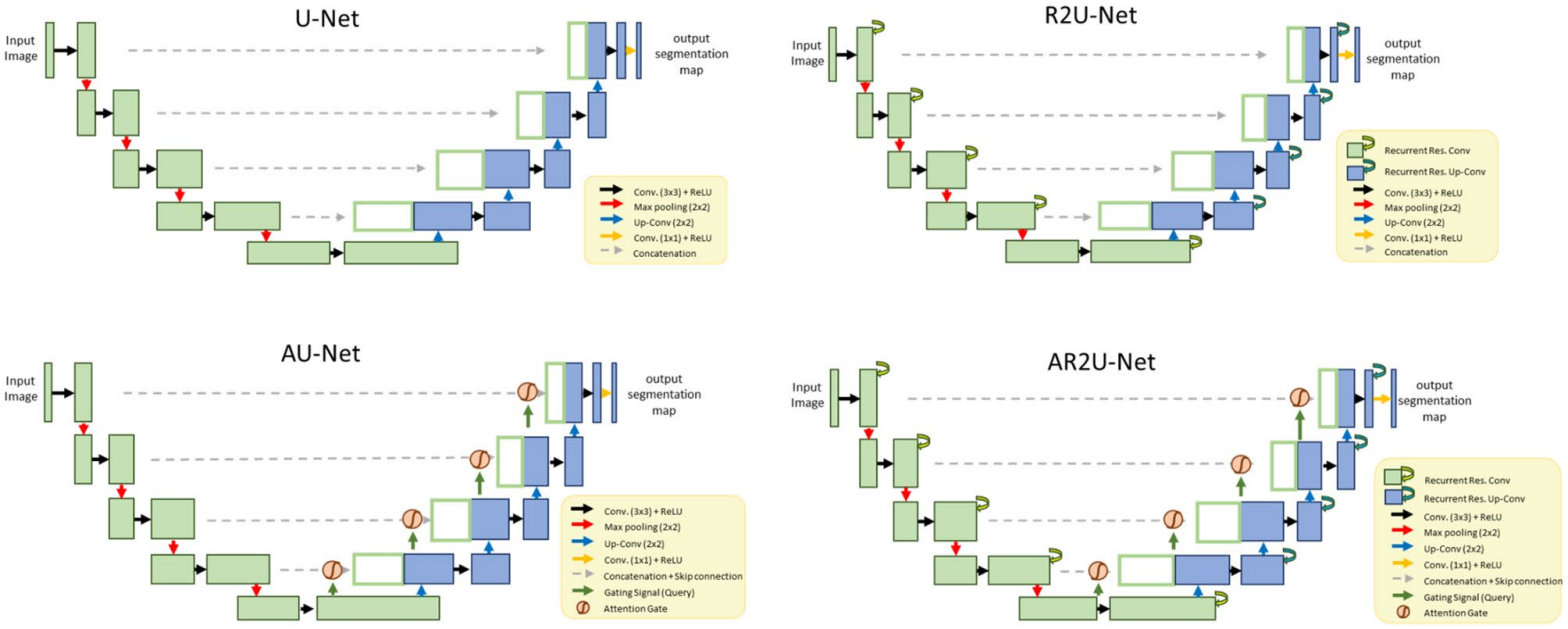
Diagrams of U-Net, R2U-Net, AU-Net and AR2U-Net architecture used for cervical spine segmentation.

For better segmentation performance, previous studies have attempted to incorporate various techniques and adjustments to the original U-Net architecture. One method used in this study with modified U-Net modules is R2U-Net, which utilizes residual layers with skip connections that forward propagate low-level and high-level information while outputs are recurred through feedback connections to store information over time, increasing performance with the same input. Another method used in this study with modified U-Net modules is AU-Net, which incorporates attention gates to suppress feature information in irrelevant regions. AR2U-Net integrates R2U-Net convolution neural networks with attention gates for suppressing irrelevant background features while enhancing segmentation performance with R2U-Net benefits. Diagrams of the four U-Net models are shown in Fig. 2.

The three U-Net networks were trained on a desktop with an Intel i9-10900 CPU, 32 GBs of RAM, and NVIDIA RTX 3060 with 12 GBs of dedicated RAM for 200 epochs, with a batch size of 3, Adam optimizer, a binary cross-entropy loss function, and a learning rate of 0.001.

### Segmentation evaluation

**Figure 3:**
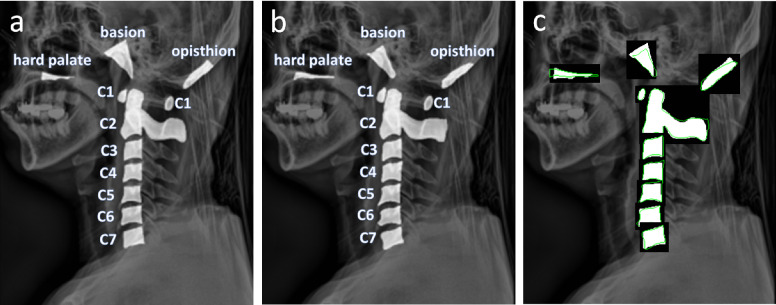
Example of overlapping predicted masks and ground truth masks for calculating dice coefficient. (**a**) Ground truth masks manually annotated. (**b**) Predicted segmentation masks (c) overlap of ground truth masks (white contours) and predicted segmentation masks (green outline) to calculate dice coefficient.

Each individual bone segment (hard palate, opisthion, basion, C1 ~ C7) was extracted and isolated from each test subject’s predicted segmentation masks and ground truth masks. The dice coefficient, HD, and MSD values of each predicted segmentation bone mask and its respective ground truth mask were calculated using the segmentation metrics python package^28^. The process was repeated for results obtained with each U-Net model. An example of predicted segmentation masks and ground truths overlapping to calculate dice coefficient is shown in Fig. 3.

### Segmentation metrics

The similarities between model predicted segmentations and manually annotated ground truths were defined through the Dice coefficient, a commonly used metric for validating segmentation accuracy and reproducibility. The Dice coefficient formula is described as 2 times the intersection of predicted masks and ground truth masks, divided by the combined number of pixels of both predicted masks and ground truth masks. The formula of the Dice coefficient is as follows:

$$DICE = \frac{2\left|P \cap G\right|}{\left|P\right| + \left|G\right|},$$

where P and G are the number of pixels in predicted segmentation and ground truth masks, respectively, and P ∩ M is the intersection of the predicted segmentation and ground truth masks.

The similarities of each boundary between predicted masks and reference masks were evaluated through HD and MSD. HD measures the largest difference between the surface distances of predicted mask and reference mask, while MSD describes the average difference between the boundaries of each surface.

## Results

The performance metrics of images tested as well as the parameters and FLOPs of the four trained U-Net models are shown in Table 1. All four models showed segmentation performance metrics of the following: Standard U-Net showed an overall average sensitivity of 89.86%, specificity of 99.52%, dice-coefficient of 89.21%, and accuracy of 99.12%. Residual U-Net showed an average sensitivity of 89.00%, specificity of 99.54%, dice-coefficient of 88.85%, and accuracy of 99.10%. Attention U-Net showed the highest average sensitivity (90.44%), dice-coefficient (89.41%), and accuracy (99.13%), but lower specificity (99.51%). Attention residual U-Net showed the highest specificity of 99.58%, but the lowest average sensitivity (87.67%), dice coefficient (88.55%), and accuracy (99.07%). AR2U-Net showed the highest computational cost of 2.454 M parameters and 0.324 FLOPs, while standard U-Net showed the lowest with 1.968 M parameters and 0.279 FLOPs.

**Table 1:** Average sensitivity, specificity, dice coefficient, accuracy, parameter count, FLOP, of U-Net, attention U-Net and residual U-Net models.

	U-Net	AU-Net	R2U-Net	AR2U-Net
Sensitivity	89.86%	**90.44%**	89.00%	87.67%
Specificity	99.52%	99.51%	99.54%	**99.58%**
Dice coefficient	89.21%	**89.41%**	88.85%	88.55%
Accuracy	99.12%	**99.13%**	99.10%	99.07%
Parameters	1.968 M	2.342 M	2.081 M	**2.454 M**
FLOPs	0.279	0.306	0.298	**0.324**

Bolded numbers represent the largest value in that category. M in parameters represents million.

Table 2 shows the average dice coefficient, HD, and MSD values of each individual bone part, obtained from segmenting each test subject’s (n = 193) X-ray image with the trained U-Net models. AU-Net showed the highest dice coefficient in most bone parts (C3, 95.30%; C4, 95.29%; C5, 95.17%; C6, 95.21%; C7, 94.66%). R2U-Net had the highest dice coefficient in segmenting the hard palate (87.76%) and the opisthion (85.71%). AR2U-Net had the highest dice coefficient in segmenting C1 (95.18%). U-Net had the lowest HD and MSD values in basion and C1 segmentations. AU-Net had the lowest HD and MSD values in C3, C4, C5, C6 and C7. AR2U-Net had the lowest HD and MSD values in hard palate, opisthion, and C2.

**Table 2:** Average dice coefficient values of each bone mask segmented by different U-Net models.

	Metric	Hard palate	Opisthion	Basion	C1	C2	C3	C4	C5	C6	C7
U-Net	DICE	87.60%	85.07%	89.29%	94.82%	**96.79%**	95.24%	95.26%	94.62%	94.06%	93.78%
	HD	16.03	13.22	**8.63**	**2.16**	4.62	2.13	2.26	2.27	2.30	2.76
	MSD	2.70	2.71	**2.03**	**0.49**	0.80	0.56	0.57	0.53	0.59	0.64
AU-Net	DICE	87.47%	85.67%	**89.69%**	95.06%	96.78%	**95.30%**	**95.29%**	**95.17%**	**95.21%**	**94.66%**
	HD	13.56	11.45	9.56	2.29	4.55	**2.07**	**2.10**	**2.24**	**2.28**	**2.67**
	MSD	2.38	2.62	2.16	0.49	0.80	**0.54**	**0.53**	**0.53**	**0.59**	**0.64**
R2U-Net	DICE	**87.76%**	**85.71%**	88.94%	94.81%	96.58%	95.00%	95.07%	95.13%	94.89%	94.60%
	HD	15.68	12.55	8.80	2.25	4.60	2.20	2.20	2.25	2.43	2.92
	MSD	2.64	2.67	2.03	0.51	0.82	0.57	0.55	0.56	0.61	0.65
AR2U-Net	DICE	86.65%	85.70%	88.49%	**95.18%**	96.48%	94.34%	94.55%	94.32%	94.47%	94.14%
	HD	**13.43**	**11.34**	9.71	2.52	**4.48**	2.21	2.34	2.44	2.37	2.91
	MSD	**2.37**	**2.60**	2.19	0.54	**0.80**	0.56	0.58	0.58	0.60	0.65

Bolded numbers represent the largest DICE value, lowest HD and MSD value.

## Discussion

For this study, four different U-Net models, standard U-Net, attention U-Net, residual U-Net, and attention residual U-Net, were trained using X-ray images of the cervical spine to segment key bone structures used to diagnose TAOD. Previous research in using machine learning models to segment the cervical spine has been limited to bones C3 and below due to clear, defined shapes and unobstructed clarity from a lack of overlap in X-rays^22,23^. As such, no methods have been yet tested for segmenting the entirety of the cervical spine in tandem with the basion, opisthion, and hard palate. All four U-Net segmentation models provided adequate segmentation performance, with average specificity and dice coefficient approaching 90%. As such, the results showed that automated segmentation of bone structures used to diagnose TAOD is possible with benefits such as rapid speed of the segmentation and less reliance on manual intervention.

**Figure 4:**
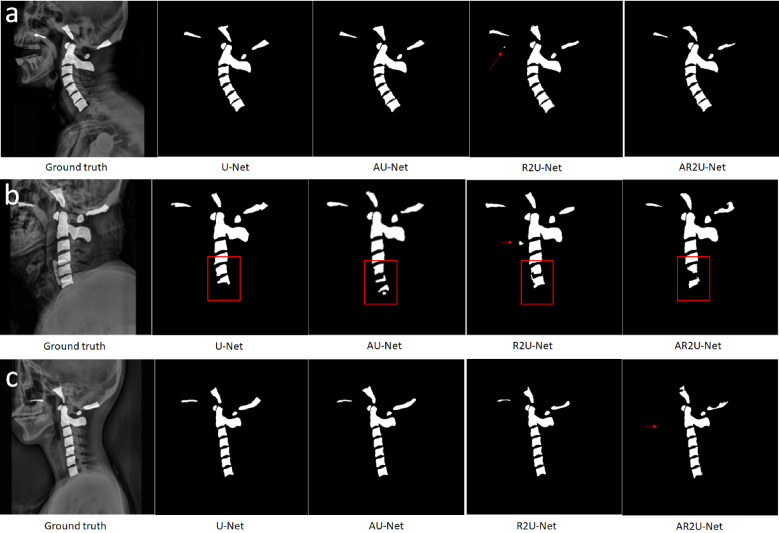
Example of errors found in U-Net predicted segmentation masks. Red arrows and red boxes refer to errors found in segmentations with certain U-Net models. (**a**) Example of a false positive in R2U-Net predicted segmentation masks. (**b**) Example of fragmented segmentations affected by spinal implants and false positives in R2U-Net predicted segmentation masks. (**c**) Example of omitted segmentation in AR2U-Net predicted segmentation masks.

The high performance of the U-Net semantic segmentation model has been a strong motivation for its utilization in many studies^24^. The encoder and decoder pathways of U-Net enable the retention of rich feature and location information, reducing the resources and time necessary to train a model with good semantic segmentation performance. The standard U-Net model, trained on 513 X-ray images with no modifications showed a sensitivity of 89.86% and an accuracy of 99.12%. In order to further optimize segmentation performance, the same dataset was trained on three different U-Net models with modifications to the standard U-Net encoder and decoder pathway. The first U-Net model trained on our dataset was AU-Net, a model that incorporates attention gates to modify feature maps and suppress features in irrelevant areas^27^. AU-Net performed the best out of the 4 models tested in average sensitivity (90.44%), dice coefficient (89.41%), and accuracy (99.13%). The second model trained on our dataset was R2U-Net, a modified U-Net neural network that incorporated recurring residual blocks that forward propagate and recur information to reduce computation resources while improving generalization. In this particular case, training R2U-Net on 513 X-ray images showed that segmentation performance suffered in all categories compared to the standard U-Net, except in specificity (99.54%). Additionally, R2U-Net introduced various false-positive errors in predicted images, segmenting areas located away from the ground truth segmentations (Fig. 4a,b). The last U-Net model trained on our dataset was AR2U-Net, a model that incorporated both attention gates and recurring residual blocks to the standard U-Net model. AR2U-Net trained with our dataset showed the lowest average sensitivity (87.67%), dice coefficient (88.55%), and accuracy (99.07%) but the highest specificity (99.58%). Segmentations with trained AR2U-Net also showed various instances with severely fragmented masks and even some cases where masks were omitted, as shown in Fig. 4b,c.

As shown in Table 1 describing computational costs, standard U-Net with no attention gates or residual blocks had the lowest number of parameters (1.968 M) and FLOPs (0.279), requiring the least number of resources and time to run out of the 4 U-Net models. R2U-Net uses slightly more parameters (2.081 M, 5.74% higher) and FLOPs (0.298, 6.8% higher) than U-Net for included residual blocks supplementing standard U-Net. However, the increased computational costs of R2U-Net results in lower sensitivity, dice coefficient and accuracy compared to U-Net results. AU-Net with attention gates uses more parameters (2.342 M) than both U-Net and R2U-Net but similar FLOPs (0.306) with R2U-Net. AR2U-Net requires the greatest number of parameters (2.454 M) and FLOPs (0.324), while providing a minor improvement in specificity and performing the worst in sensitivity, dice coefficient and accuracy. Purely based on model metrics, AR2U-Net were the least efficient in terms of computational power, requiring 16.13% FLOPs over standard U-Net for worse sensitivity, dice coefficient and accuracy. On the contrary, AU-Net showed minor improvements over standard U-Net in sensitivity, dice coefficient and accuracy at the costed of 9.68% increased FLOPs.

**Figure 5:**
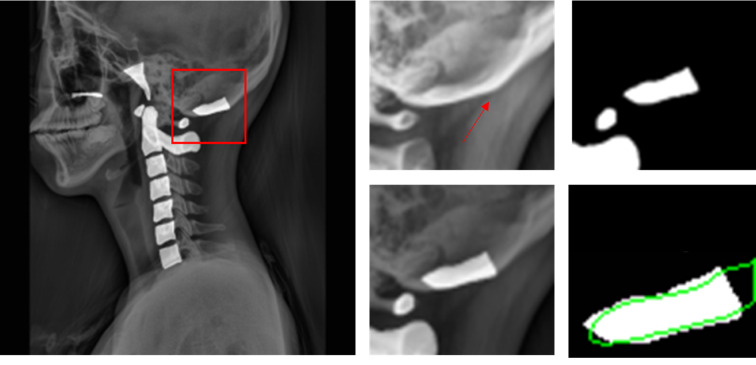
An example of obfuscated boundaries of an opisthion’s posterior end. Red arrow points to the ground truth boundary of the opisthion’s posterior end. The green outline shows an example of an AU-Net predicted opisthion mask, which extends further beyond the ground truth.

**Figure 6:**
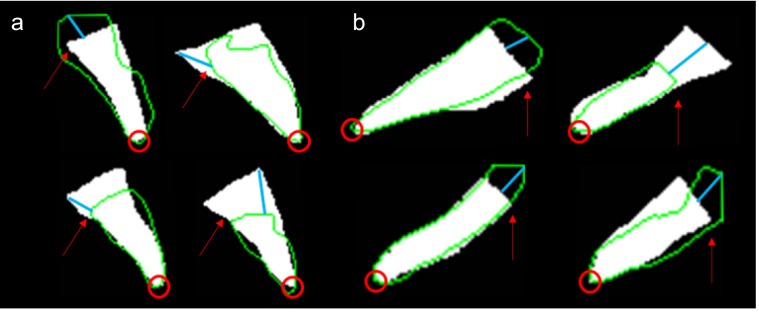
Example of masks that show poor dice coefficient, high HD, and MSD but with high accuracy in regions used for measuring metrics for diagnosing TAOD. Green outlines refer to AU-Net segmentation masks, white masks refer to ground truths. Blue lines refer to a visual depiction of how HD can be calculated. Red arrows refer to posterior ends of cranial bones with inaccurate posterior boundaries but are not used in determining TAOD metrics. Red circles refer to anterior ends that are used for determining TAOD metrics. (**a**) Basion masks. (**b**) Opisthion masks.

The average dice coefficient, HD, and MSD values of each bone mask segmented with the 4 different U-Net models were compared for evaluation. Dice coefficient described how well the surfaces of each segmentation overlapped with each other, while HD and MSD values described how well the predicted segmentation matched its boundaries with the reference mask. Results showed standard U-Net had the highest dice coefficient when segmenting C2 (96.79%), and the lowest HD and MSD value in basion and C1 segmentations. R2U-Net had the highest dice coefficient when segmenting the the hard palate (87.76%) and opisthion (85.71%). AU-Net showed the highest dice coefficient in segmenting the basion (89.69%), C3 (95.30%), C4 (95.29%), C5 (95.17%), C6 (95.21%), C7 (94.66%). AU-Net also showed the lowest HD and MSD values in C3, C4, C5, C6, and C7 segmentations. AR2U-Net showed the highest dice coefficient in C1 segmentations (95.18%) and the lowest HD and MSD in hard palate, opisthion and C2 segmentations. Compared to bones C1 through C7, the hard palate, opisthion, and basion showed noticeably lower dice coefficient values and higher HD and MSD in all U-Net models, partially due to vague boundaries from X-ray blurs making some segmentations dependent on human judgment as shown in Fig. 5. At a first glance, it would seem that a noticeably lower hard palate HD (13.43) and MSD (2.37) in AR2U-Net over standard U-Net hard palate HD (16.03) and MSD (2.70) shows the advantages of AR2U-Net despite the lower dice coefficient (86.65% compared to 87.60%) due to how TAOD metrics are mainly calculated using the boundaries of segmentations. However, the inconsistent boundary selection of the three cranial bones: the hard palate, opisthion, and the basion, are usually isolated to the posterior ends of each bone structure, areas that are not considered for calculating TAOD related metrics. For example, the TAOD metrics such as the BDI, which takes the distance between the inferior (lowest) point of the basion and the closest point of superior C2^9^ as shown in Fig. 1b. As such, evaluating each U-Net model’s performance in accurately determining TAOD metrics may not be consistent with dice coefficient, HD, or MSD values as visualized in Fig. 6. The dice coefficient, HD, and MSD values of bones C1 through C7 were mostly similar among the four U-Net models, due to unobfuscated boundaries making segmentation and prediction consistent. However, minor variations of metrics were noticeable in C5, C6, and C7 bones, in part due to a number of subjects with spinal implants commonly located in C5, C6, and C7 bones, as shown in Fig. 4b.

There are some limitations to consider for this study. First, as previously mentioned, the manual segmentation of ground truths is significantly influenced by human judgment. Regions with the highest likelihood for error are mainly located at the posterior ends of cranial bones where boundaries are heavily obfuscated by bone stacking effects in X-rays. While it may take significant resources to do so, making additional redundant ground truths segmented by a multitude of trained professionals can help improve consistency. Second, it is difficult to conclude which U-Net model is the most useful in determining TAOD metrics due to only measuring segmentation performance. As previously mentioned, segmentations of cranial bones with low dice coefficient values or high HD and MSD values can still show accurate measurements of TAOD metrics. Third, the patterns shown in our segmentation results may differ depending on the training or testing data used. Since our data used to train U-Net models was moderately consistent, it is possible that each U-Net model trained or tested on data with heavily fractured bones or with spinal implants can show different results.

To the best of our knowledge, this study was the first to train U-Net and 3 other U-Net variant models for segmenting the cervical spine, as well as the 3 cranial bones, hard palate, opisthion, and the basion, areas in which are involved in calculating metrics used for diagnosing TAOD. Our methods shown in this study present a potential avenue for automated rapid diagnosis of TAOD using X-rays. Additionally, we show that modifications to the standard U-Net model can be pursued to potentially further improve cervical spine segmentation performance.

## Data Availability

The U-Net models and code generated for this study are available from the corresponding author on responsible request.
