# Author Correction: Evaluation of U-Net models in automated cervical spine and cranial bone segmentation using X-ray images for traumatic atlanto-occipital dislocation diagnosis

**DOI:** 10.1038/s41598-023-29770-y

**Published:** 2023-02-14

**Authors:** Jae-Hyuk Shim, Woo Seok Kim, Kwang Gi Kim, Gi Taek Yee, Young Jae Kim, Tae Seok Jeong

**Affiliations:** 1 Department of Biomedical Engineering, Gil Medical Center, Gachon University College of Medicine, Incheon, Korea; 2 Department of Traumatology, Gil Medical Center, Gachon University College of Medicine, Incheon, Korea; 3 Department of Neurosurgery, Gil Medical Center, Gachon University College of Medicine, Incheon, Korea

Correction to: *Scientific Reports* https://doi.org/10.1038/s41598-022-23863-w, published online 12 December 2022

In the original version of this Article Jae-Hyuk Shim and Woo Seok Kim were omitted as equally contributing authors. In addition, the Author Contributions section in this Article was incomplete.

“All authors reviewed and approved the manuscript. All authors made significant contributions to the manuscript. K.G.K., Y.J.K., G.T.Y. designed and supervised the research. W.S.K., T.S.J. provided data and guidance for research. J.H.S. performed the experiments and wrote the main manuscript text.”

now reads:

“All authors reviewed and approved the manuscript. All authors made significant contributions to the manuscript. K.G.K., Y.J.K., G.T.Y. designed and supervised the research. W.S.K., T.S.J. provided data and guidance for research. J.H.S. performed the experiments and wrote the main manuscript text. J.H.S and W.S.K made equal contributions to the manuscript.”

Furthermore, the Acknowledgements section in the original version of this Article was incomplete.

“This work was supported by the GRRC program of Gyeonggi province. [GRRC-Gachon2020(B01), AI-based Medical Image Analysis, and by the Gachon University (GCU-202205980001).”

now reads:

“This work was supported by the GRRC program of Gyeonggi province. [GRRC-Gachon2020(B01), AI-based Medical Image Analysis, and by the Gachon University (GCU-202205980001) and by the MSIT (Ministry of Science and ICT), Korea, under the ITRC (Information Technology Research Center) support program (IITP-2022-2017-0-01630) supervised by the IITP (Institute for Information & communications Technology Promotion).”

The original Article has been corrected.

